# Summer Sours

**Published:** 1859-06

**Authors:** 


### SUMMER SOURS.

Physiological research has fully established the fact that acids promote the separation of the bile from the blood, which is then passed from the system, thus preventing fevers, the prevailing diseases of summer. All fevers are "billious," that is, the bile is in the blood. Whatever is antagonistic of fever, is cooling. It is a common saying that fruits are "cooling," and also berries of every description; it is because the acidity which they contain aids in separating the bile from the blood, that is, aids in purifying the blood. Hence the great yearning for greens and lettuce, and salads in the early spring, these being eaten with vinegar; hence also the taste for something sour, for lemonades, on an attack of fever.

But this being the case, it is easy to see, that we nullify the good effects of fruits and berries, in proportion as we eat them with sugar, or even sweet milk, or cream. If we eat them in their natural state, fresh, ripe, perfect, it is almost impossible to eat too many, to eat enough to hurt us, especially if we eat them alone, not taking any liquid with them whatever. Hence also is buttermilk or even common sour milk promotive of health in summer time. Sweet milk tends to billousness in sedentary people, sour milk is antagonistic. The Greeks and Turks are passionately fond of sour milk. The shepherds use rennet, and the milk dealers alum to make it sour the sooner. Buttermilk acts like watermelons on the system.

